# The prognostic and diagnostic significance of inflammatory markers TNF-α, IL-6, and IFN-γ in evaluating disease severity in diabetic foot infection

**DOI:** 10.3389/fcimb.2025.1606612

**Published:** 2025-07-03

**Authors:** Shuo Wang, Lei Gao, Xinyuan Qin, Tianbo Li, Jiangning Wang, Huimin Xie

**Affiliations:** ^1^ Department of Orthopedics, Beijing Shijitan Hospital, Capital Medical University, Beijing, China; ^2^ Department of Rehabilitation, The First Medical Center of Chinese People's Liberation Army General Hospital, Beijing, China

**Keywords:** diabetic foot infection, TNF-α, IL-6, IFN-γ, severity, prognosis

## Abstract

**Objective:**

To investigate the relationship between TNF-α, IL-6, and IFN-γ serum levels and the severity of infection and prognosis in patients with diabetic foot infection (DFI).

**Methods:**

A total of 144 patients diagnosed with diabetic foot at our hospital from January 2020 to December 2023 were enrolled in the study. Patients were divided into an infection group (70 cases) and a non-infection group (74 cases) based on the presence of infection. The infection group was further categorized into mild (29 cases), moderate (18 cases), and severe infection (23 cases) subgroups according to infection severity. Serum levels of TNF-α, IL-6, and IFN-γ in DFI patients were analyzed, and their predictive value for treatment outcomes was evaluated

**Results:**

Serum levels of TNF-α, IL-6, and IFN-γ were significantly higher in the infection group than in the non-infection group (P<0.05). Moreover, there were significant differences in TNF-α, IL-6, and IFN-γ levels among patients with mild, moderate, and severe infections (P<0.05). ROC curve analysis demonstrated that the area under the curve (AUC) for the combined detection of TNF-α, IL-6, and IFN-γ in assessing DFI severity was 0.855, which was significantly higher than that of TNF-α (0.811), IL-6 (0.793), and IFN-γ (0.764) (P<0.05). Furthermore, serum levels of TNF-α, IL-6, and IFN-γ were significantly higher in the poor prognosis group than in the good prognosis group (P<0.05). ROC curve analysis showed that the AUC for predicting poor prognosis in DFI patients was 0.926 when TNF-α, IL-6, and IFN-γ were combined, which was significantly higher than that of TNF-α (0.849), IL-6 (0.834), and IFN-γ (0.809) (P<0.05).

**Conclusion:**

Serum levels of TNF-α, IL-6, and IFN-γ are elevated in DFI patients and are closely associated with infection severity and prognosis. The combined detection of these three inflammatory factors can serve as a predictive indicator for infection severity and poor prognosis in DFI patients.

## 1 Introduction

Diabetic foot is one of the most common complications of diabetes, primarily caused by neuropathy and vascular lesions induced by prolonged hyperglycemia (Jeffcoate et al., 2018). Clinical data indicate that the incidence of diabetic foot is rising annually, likely due to the increasing number of diabetic patients and the trend of population aging (Lavery et al., 2019). Chronic hyperglycemia damages the nervous and vascular systems, leading to reduced sensation in the feet and impaired blood circulation, which significantly increases the risk of foot ulcers and infections. In severe cases, this can result in gangrene and necessitate amputation (Castiglioni et al., 2017; Lauri et al., 2020; Lavery et al., 2020). Currently, the amputation rate among diabetic foot patients is approximately 1.1%, with infection playing a crucial role in the onset and progression of diabetic foot. It is also a major cause of disability and mortality in these patients (Lipsky et al., 2004; Pitocco et al., 2019). More than 50% of diabetic foot wounds exhibit infection at an early stage. Therefore, clinical efforts should focus on preventing and controlling DFI and identifying convenient and sensitive biomarkers for assessing infection severity and prognosis to guide clinical diagnosis and treatment.

Tumor necrosis factor-alpha (TNF-α) is a potent pro-inflammatory cytokine produced by monocytes, T lymphocytes, and macrophages, playing a crucial role in inflammatory responses and cellular immunity (Wang et al., 2021). Studies have shown that TNF-α expression is significantly upregulated and prolonged during the chronic wound healing process of infected diabetic foot ulcers (Dhamodharan et al., 2019). Interleukin-6 (IL-6) is a key inflammatory mediator involved in inflammatory responses and immune regulation (Pena-Duran et al., 2025). Research has indicated that IL-6 levels are positively correlated with disease severity in patients with type 2 diabetes, and excessive IL-6 may impair tissue repair capacity (Nicchio et al., 2025). Interferon-gamma (IFN-γ), primarily secreted by T helper 1 (Th1) cells, is a key inflammatory cytokine involved in the pathogenesis of diabetes and is closely associated with secondary infections in diabetic foot ulcers (Umapathy et al., 2018; Amin et al., 2020).

TNF-α, IL-6, and IFN-γ play crucial roles in DFI and may reflect different pathophysiological mechanisms and clinical significance. Investigating the changes in these three biomarkers in DFI patients and their association with disease progression can enhance the understanding of DFI pathophysiology and provide valuable insights for early diagnosis and individualized treatment strategies. This study aims to examine the peripheral blood levels of TNF-α, IL-6, and IFN-γ in DFI patients and analyze their relationship with infection severity and prognosis, thereby offering diagnostic evidence for the early identification and personalized treatment of DFI.

## 2 Study subjects and methods

### 2.1 Study subjects

This study enrolled 144 patients diagnosed with diabetic foot who were treated at Beijing Shijitan Hospital from January 2020 to December 2023. The inclusion criteria were as follows: (1) meeting the diagnostic criteria for type 2 diabetes; (2) meeting the diagnostic criteria for diabetic foot; (3) actively cooperating with treatment and demonstrating good adherence; (4) age between 18 and 80 years; and (5) providing informed consent and signing the consent form. The exclusion criteria included: (1) type 1 diabetes; (2) coexisting diabetes-related complications; (3) presence of other infectious diseases; (4) severe cardiac, hepatic, or renal dysfunction; (5) malignancies; (6) long-term use of corticosteroids or immunosuppressants; and (7) psychiatric disorders. Based on the presence of infection, the patients were categorized into an infection group (n=70) and a non-infection group (n=74). This study was approved by the Ethics Committee of Beijing Shijitan Hospital, Capital Medical University (IIT2025-002-002).

### 2.2 Data collection

Upon hospital admission, demographic and clinical data, including gender, age, diabetes history, and body mass index (BMI). The determination of infection severity was based on the Wagner classification (Lipsky et al., 2020), categorized as mild (grades 0-2), moderate (grade 3), and severe (grades 4-5). According to the 4-week follow-up outcomes of DFI patients, those who achieved wound healing were classified into the good prognosis group, whereas patients with unhealed wounds, amputations, or death were classified into the poor prognosis group. For all patients, 3 mL of venous blood was collected in the early morning under fasting conditions and centrifuged at 3,000 r/min for 5 minutes. The supernatant was then stored at -80°C for subsequent analysis. The levels of TNF-α, IL-6, and IFN-γ were measured using a sandwich ELISA assay (Invitrogen, Carlsbad, California, USA). The intra- and inter-assay coefficient of variation (CV) was <10%, with concentrations expressed in ng/L. All procedures were performed strictly according to the manufacturer’s instructions.

### 2.3 Statistical analysis

Data analysis was performed using SPSS 23.0 statistical software. Normally distributed quantitative data were expressed as mean ± standard deviation ( $\bar{\text{x}}$ ± s), and comparisons between groups were conducted using the t-test. One-way analysis of variance (ANOVA) was used for comparisons among multiple groups, followed by the Student-Newman-Keuls (SNK-q) test for pairwise comparisons. Categorical data were presented as counts (n) or percentages (%) and analyzed using the chi-square (χ²) test. The receiver operating characteristic (ROC) curve was used to evaluate the predictive value of serum TNF-α, IL-6, and IFN-γ levels in assessing infection severity and prognosis in DFI patients. For the analysis of ROC curves, we compared the diagnostic performance of three biomarkers. To account for multiple comparisons, Bonferroni correction was applied, adjusting the significance level to α/m, where m is the number of comparisons (m = 3). Thus, the corrected significance threshold was set to α = 0.05/3 = 0.0167. A p-value below this threshold was considered statistically significant, indicating that the biomarker’s diagnostic performance was significantly different from the null hypothesis. The area under the curve (AUC) comparisons were conducted using the Z-test, with statistical significance set at P<0.05.

## 3 Results

### 3.1 Comparison of general characteristics and serum TNF-α, IL-6, and IFN-γ levels between infection and non-infection groups

The general clinical characteristics, including gender, age, diabetes history, and BMI, were compared between the infection and non-infection groups. No significant differences were observed between the two groups (P>0.05), indicating comparability (**Table 1**). However, serum levels of TNF-α, IL-6, and IFN-γ were significantly higher in the infection group than in the non-infection group, with statistically significant differences (P<0.05) (**Table 2**).

**Table 1:** Comparison of baseline characteristics between the two patient groups.

Characteristics	Infection(n=70)	Non-Infection (n=74)	P value
Gender (Male/Female)	45/25	48/36	0.537
Age(y)	57.28 ± 7.83	59.66 ± 9.45	0.367
Diabetes history (y)	13.87 ± 4.31	12.89 ± 5.03	0.285
BMI (kg/m^2^)	24.06 ± 2.95	23.86 ± 2.15	0.452

**Table 2:** Comparison of serum TNF-α, IL-6, and IFN-γ levels between the two patient groups.

Indicator	Infection(n=70)	Non-Infection(n=74)	t value	P value
TNF-α (ng/L)	36.76 ± 6.05	23.78 ± 7.87	7.897	<0.01
IL-6 (ng/L)	58.53 ± 7.22	36.75 ± 6.89	6.743	<0.01
IFN-γ (ng/L)	8.33 ± 2.82	5.75 ± 1.98	9.527	<0.01

### 3.2 Comparison of serum TNF-α, IL-6, and IFN-γ levels among patients with different infection severity and prognosis

Patients were classified into mild infection (n=29), moderate infection (n=18), and severe infection (n=23) groups based on infection severity. According to our p retrospective power analysis, the study achieved a statistical power of 82.5% at the α = 0.05 significance level, indicating a statistically reliable result within the context of the observed effect size. Serological analysis revealed a progressive increase in TNF-α, IL-6, and IFN-γ levels with increasing infection severity, with statistically significant differences among the groups (P<0.05) (**Table 3**). Additionally, patients were categorized into a good prognosis group (n=37) and a poor prognosis group (n=107) based on clinical outcomes. Serum levels of TNF-α, IL-6, and IFN-γ were significantly lower in the good prognosis group compared to the poor prognosis group, with statistically significant differences (P<0.05) (**Table 4**).

**Table 3:** Comparison of serum levels of TNF-α, IL-6, and IFN-γ among patients with different severities of infection.

Indicator	Infection severity			F value	P value
	Mild (n=29)	Moderate (n=18)	Severe (n=23)		
TNF-α (ng/L)	29.68 ± 7.45	35.94 ± 5.88	39.85 ± 6.07	6.122	0.013
IL-6 (ng/L)	50.38 ± 5.12	56.77 ± 8.02	60.91 ± 7.82	7.982	0.021
IFN-γ (ng/L)	4.83 ± 1.91	7.62 ± 3.72	10.91 ± 4.78	10.817	<0.01

**Table 4:** Comparison of serum TNF-α, IL-6, and IFN-γ levels between the good and poor prognosis groups.

Indicator	Prognosis		t value	P value
	Good (n=37)	Poor(n=107)		
TNF-α (ng/L)	21.63 ± 5.95	40.15 ± 9.92	5.751	<0.01
IL-6 (ng/L)	28.31 ± 8.21	53.57 ± 8.93	8.369	<0.01
IFN-γ (ng/L)	4.83 ± 1.26	9.05 ± 3.87	6.972	<0.01

### 3.3 Diagnostic value of serum TNF-α, IL-6, and IFN-γ levels in assessing infection severity and prognosis in DFI patients

**Figure 1:**
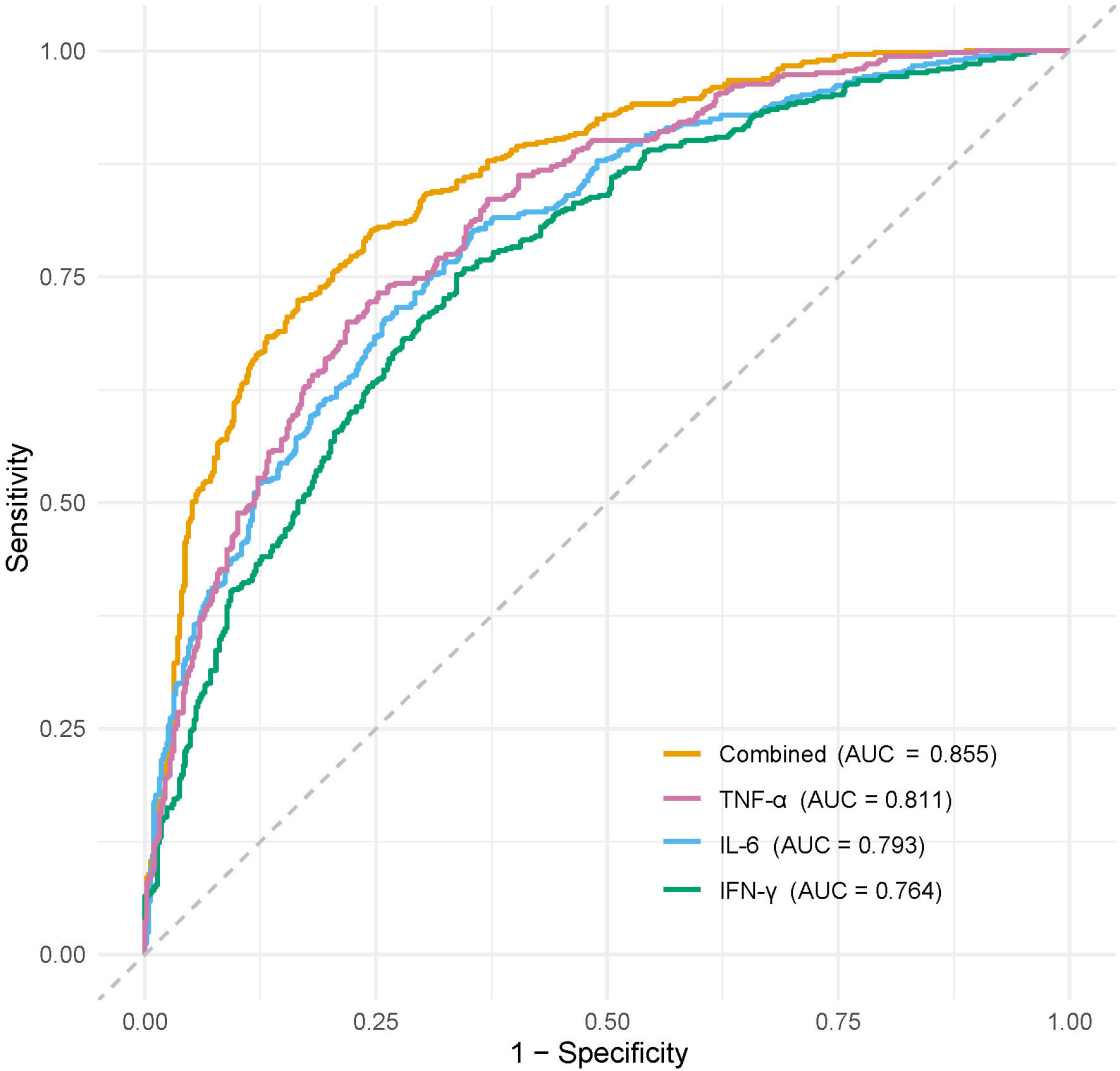
ROC curve of serum TNF-α, IL-6, and IFN-γ levels in diagnosing the severity of DFI patients.

**Figure 2:**
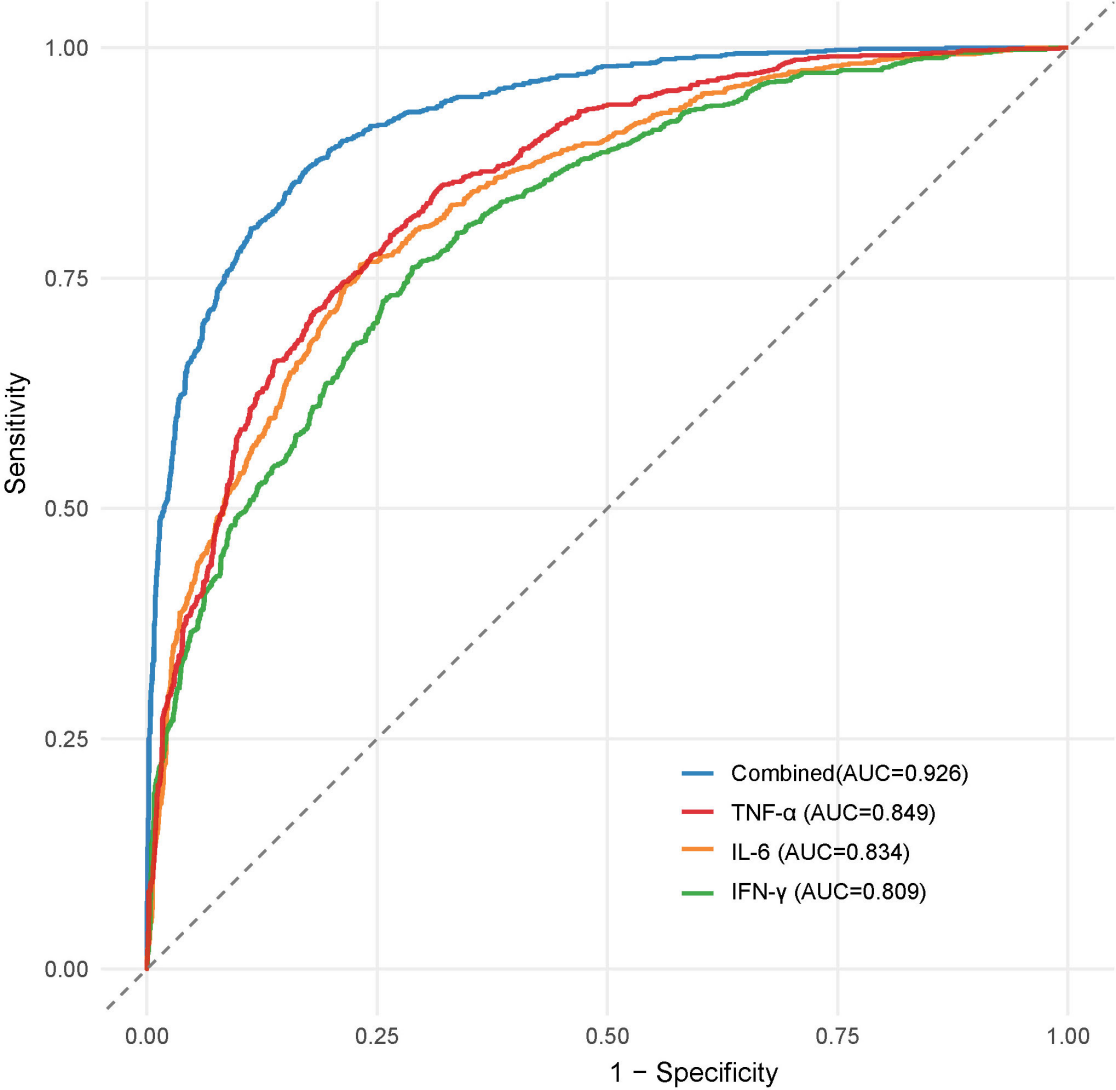
ROC curve of serum TNF-α, IL-6 and IFN-γ levels in diagnosing the prognosis of DFI patients.

For infection severity diagnosis, ROC curve analysis demonstrated that serum TNF-α, IL-6, and IFN-γ levels had diagnostic value in assessing DFI infection severity, with AUC values of 0.811(95%CI: 0.734-0.927), 0.793(95%CI: 0.705-0.857), and 0.764(95%CI: 0.699-0.839), respectively. Combined detection improved diagnostic efficacy, yielding an AUC of 0.855(95%CI: 0.814-0.984) (**Table 5**; **Figure 1**). Regarding prognosis prediction, serum TNF-α, IL-6, and IFN-γ levels showed strong predictive value for DFI prognosis, with AUC values of 0.849(95%CI: 0.791-0.909), 0.834(95%CI: 0.743-0.898), and 0.809(95%CI: 0.715-0.873), respectively. The combined detection further enhanced predictive performance, achieving an AUC of 0.926(95%CI: 0.856-0.992) (**Table 6**; **Figure 2**).

**Table 5:** Diagnostic value of serum TNF-α, IL-6, and IFN-γ in assessing the severity of infection.

Indicator	AUC	95%CI	Sensitivity(%)	Specificity(%)
TNF-α	0.811	0.734-0.927	79.83	83.21
IL-6	0.793	0.705-0.857	75.34	73.98
IFN-γ	0.764	0.699-0.839	70.28	74.43
Combined	0.855	0.814-0.984	84.77	88.63

**Table 6:** Diagnostic value of serum TNF-α, IL-6, and IFN-γ in predicting the prognosis of DFI patients.

Indicator	AUC	95%CI	Sensitivity(%)	Specificity(%)
TNF-α	0.849	0.791-0.909	80.35	78.52
IL-6	0.834	0.743-0.898	79.93	85.74
IFN-γ	0.809	0.715-0.873	81.44	84.38
Combined	0.926	0.856-0.992	86.36	90.05

## 4 Discussion

Due to impaired immune function in diabetic patients, vascular lesions can easily trigger ischemic necrosis. Additionally, sensory and motor neuropathy contribute to the development of diabetic foot. Once foot ulcers form, pathogenic microorganisms can readily invade deep tissues, leading to infections (Nauriyal and Byers, 2025). Infection is not only a crucial factor in exacerbating diabetic foot but also a leading cause of amputation. The hyperglycemic environment and malnutrition in diabetic patients create ideal conditions for bacterial growth and proliferation. Meanwhile, impaired leukocyte function further increases the risk of local soft tissue infections. Epidemiological studies indicate that the incidence of DFI has been rising annually and is projected to reach 7.7% by 2030 (Bakhtiyari et al., 2021). Therefore, accurately assessing DFI severity and implementing early intervention is essential for improving patient outcomes (Alvarez et al., 2020). Current conventional infection markers have limitations in assessing DFI severity, highlighting the importance of identifying new infection biomarkers and utilizing combined detection strategies for clinical applications. This study primarily investigates the correlation between inflammatory cytokines TNF-α, IL-6, and IFN-γ with DFI and further analyzes their relationship with infection severity, aiming to provide clinical guidance for early intervention, thereby improving patient prognosis and quality of life.

In recent years, the role of inflammation in diabetes and its complications has garnered increasing attention. Studies suggest that diabetic patients experience a chronic low-grade inflammatory state, and elevated levels of inflammatory cytokines are closely associated with the onset and progression of diabetes and its complications. For example, IL-6 levels are positively correlated with the severity of type 2 diabetes, and elevated IL-6 impairs tissue repair, leading to poor healing of diabetic foot ulcers. T lymphocytes play a crucial role in balancing inflammatory responses and microbial tolerance (Kronsteiner et al., 2019; Moura et al., 2019). TNF-α, a key cytokine in the Th1 signaling pathway, promotes the expression of intercellular adhesion molecules, facilitating the migration of activated neutrophils and T cells from the epidermis to the dermis. Additionally, TNF-α mediates the Th17 signaling pathway by interacting with dendritic and tissue cells, inducing IL-17 and IL-23 secretion to maintain tissue homeostasis. However, excessive TNF-α release may cause pathological damage, disrupting immune balance (Wang et al., 2021). Studies have shown that peripheral blood monocyte activity is enhanced in DFI patients, leading to increased TNF-α levels (Sayed and Mahmoud, 2016). IFN-γ, secreted by activated T cells, possesses antiviral and cell growth-inhibitory functions. In diabetic patients, major histocompatibility complex class I (MHC-I) expression in pancreatic β-cells is upregulated, making them more susceptible to T-cell cytotoxicity. IFN-γ promotes diabetes development by inducing aberrant MHC expression. Moreover, IFN-γ directly inhibits β-cell proliferation and mediates pancreatic cell damage through multiple pathways (Samuel et al., 2019). Studies suggest that improvements in inflammatory biomarkers such as serum IFN-γ levels and the neutrophil-to-lymphocyte ratio are closely related to glycemic control (Abdel-Moneim et al., 2019). During the wound healing process of DFI, these inflammatory factors work synergistically, affecting immune defense in the early stages of inflammation and tissue repair in the later stages. The sustained high expression of TNF-α and IFN-γ may lead to delayed inflammation and the formation of chronic wounds, while the overexpression of IL-6 may promote the expression of chemokines, attracting macrophages to the infection site, thereby maintaining a chronic inflammatory state and delaying wound healing (Weigelt et al., 2009; Lee et al., 2019). Therefore, precisely regulating the levels of these inflammatory factors may be a key strategy for improving the treatment outcomes of DFI.

The results of this study indicate that serum levels of TNF-α, IL-6, and IFN-γ were significantly elevated in DFI patients compared to diabetic foot patients without infection, and their levels positively correlated with infection severity. In other words, the higher the levels of these inflammatory factors, the more severe the infection. Additionally, the expression levels of these cytokines significantly differed between patients with different prognoses. Patients in the good prognosis group had significantly lower serum TNF-α, IL-6, and IFN-γ levels than those in the poor prognosis group. These findings suggest that abnormal elevations in TNF-α, IL-6, and IFN-γ levels are closely associated with DFI severity and poor prognosis. Furthermore, ROC curve analysis demonstrated that serum TNF-α, IL-6, and IFN-γ levels have good predictive value for DFI infection severity and prognosis, and their combined detection further improves predictive accuracy.

The levels of inflammatory factors have significant clinical application value in the early diagnosis, disease assessment, and personalized treatment of DFI. IL-6 is usually significantly elevated in DFI patients, and its sustained high levels may indicate a chronic inflammatory state, while excessive expression of TNF-α is associated with tissue necrosis and worsening of lesions (Falanga, 2005). In addition, IFN-γ plays a key role in assessing the severity of infection; its deficiency may reflect an immunosuppressive state, while overexpression may lead to uncontrolled inflammation and delayed wound healing (Donath, 2014; Abdel-Moneim et al., 2019). In the field of precision medicine, monitoring inflammatory factors can guide personalized treatment strategies. For example, anti-TNF-α drugs may be suitable for patients with excessive inflammation, while IL-6 antagonists can be used to reduce tissue damage caused by chronic inflammation (Donath, 2014). Moreover, biomarker-driven treatment models, such as the combined detection of TNF-α, IL-6, and IFN-γ, to optimize the use of antibiotics or immunomodulatory agents, help improve the precision of DFI diagnosis and treatment, avoiding over- or under-treatment. In the future, by integrating multi-omics data (such as genomics, proteomics, and metabolomics), predictive models or artificial intelligence algorithms based on individual inflammatory factor profiles may further enhance early screening and personalized intervention for DFI.

In the diagnosis and prognostic assessment of diabetic foot infections, inflammatory biomarkers such as TNF-α, IL-6, and IFN-γ have been extensively studied and show promise in reflecting disease activity when compared to traditional biomarkers like CRP and WBC. Although traditional biomarkers are widely used in clinical practice due to their ease of detection and low cost, inflammatory markers such as TNF-α, IL-6, and IFN-γ offer a more accurate reflection of both local and systemic immune responses, thus being considered of higher diagnostic and prognostic value in certain studies. However, the detection of these newer biomarkers involves higher costs and requires more sophisticated laboratory equipment and technical expertise, which may limit their widespread application in resource-limited settings. Given the feasibility of detection, the routine use of TNF-α, IL-6, and IFN-γ may face challenges, especially when integrated with existing clinical severity scoring systems, such as the Wagner score, which may provide more biological insight. Therefore, while these biomarkers have potential clinical applicability, their integration into routine diagnostic workflows needs further validation, particularly in low-resource environments, where combining them with traditional biomarkers may be more feasible to improve diagnostic accuracy and treatment decision-making.

In conclusion, serum TNF-α, IL-6, and IFN-γ levels are significantly elevated in DFI patients, and all three cytokines serve as potential biomarkers for assessing infection severity and prognosis. Moreover, combined detection enhances diagnostic accuracy. However, since this study only included single-center clinical data, the sample size and types of statistical data were limited, which may result in insufficient statistical power and make it difficult to generalize the findings to a broader population. Furthermore, as the study was conducted at a single research center, patient sources and treatment approaches may have regional or institution-specific biases, which could affect the external validity of the results. In the future, we plan to conduct multi-center studies, which will include a more diverse population, enhance the generalizability and reliability of the findings, and improve statistical power to draw more convincing conclusions. Additionally, we will further expand the sample size and continue to validate the reliability and practicality of the combined detection of TNF-α, IL-6, and IFN-γ in clinical practice, while exploring the specific mechanisms of TNF-α, IL-6, and IFN-γ in the development and progression of DFI to optimize diagnostic and therapeutic strategies for DFI.

## Data Availability

The original contributions presented in the study are included in the article/supplementary material. Further inquiries can be directed to the corresponding author.
